# Combined multiomics analysis reveals the mechanism of CENPF overexpression-mediated immune dysfunction in diffuse large B-cell lymphoma *in vitro*

**DOI:** 10.3389/fgene.2022.1072689

**Published:** 2022-12-30

**Authors:** Dan Yang, Jia Wang, Mingqiu Hu, Feng Li, Feifei Yang, Youcai Zhao, Yanli Xu, Xuezhong Zhang, Lijun Tang, Xiuqun Zhang

**Affiliations:** ^1^ Department of Hematology, Nanjing First Hospital, Nanjing Medical University, Nanjing, Jiangsu, China; ^2^ Department of Nuclear Medicine, The First Affiliated Hospital of Nanjing Medical University, Nanjing, Jiangsu, China; ^3^ Department of Pathology, Nanjing First Hospital, Nanjing Medical University, Nanjing, Jiangsu, China

**Keywords:** centrimeric protein F, diffuse large B-cell lymphoma, proteome, tumor escape, ubiquitination

## Abstract

Diffuse large B-cell lymphoma (DLBCL) is one of the most common aggressive B-cell lymphomas with significant heterogeneity. More than half of patients are cured, but 40%–45% still face relapse or develop drug resistance, and the mechanism is not yet known. In this study, Centrimeric protein F (CENPF) overexpression was found in several DLBCL patients with relapsed or refractory disease compared to patients with complete remission. Thus, the human DLBCL cell line SU-DHL-4 was chosen for this study, and CENPF was upregulated in that cell line by using an adenovirus *in vitro*. Mass spectrometry-based quantitative proteome analysis was first performed, and the results showed that the expression levels of various proteins were increased when CENPF was upregulated, and these proteins are mainly involved in cellular processes, biological regulation, immune system processes and transcriptional regulator activity. Bioinformatics data analysis revealed that the main enriched proteins, including UBE2A, UBE2C, UBE2S, TRIP12, HERC2, PIRH2, and PIAS, were involved in various ubiquitin-related kinase activities and ubiquitination processes. Thus, ubiquitinome analysis was further performed, and the results demonstrated that proteins in many immune-related cellular pathways, such as natural killer cell-mediated cytotoxicity, the T-cell receptor signaling pathway and the B-cell receptor signaling pathway, were significantly deubiquitinated after CENPF was upregulated in DLBCL cells. Furthermore, TIMER2.0 was also used to reveal the association between CENPF and immune infiltration in DLBCL. The results showed that CENPF expression was positively correlated with CD8^+^ T cells, NK cells and B lymphocytes in DLBCL samples but negatively correlated with regulatory T cells. Aberrant activation of CENPF may induce immune dysregulation in DLBCL cells by mediating protein deubiquitination in various immune signaling pathways, which leads to tumor escape of DLBCL, but further experimental validation is still needed.

## Introduction

Diffuse large B-cell lymphoma (DLBCL) is an aggressive B-cell lymphoma. It is the most common type of non-Hodgkin lymphoma (NHL), which has significant heterogeneity with different prognoses for patients with different subtypes (Smith et al., 2015; Cazzola, 2016). DLBCL can be divided into the germinal center (GCB) type and non-germinal center (non-GCB) type according to the cell of origin (COO) classification, where the activated B cell (ABC) subtype of non-GCB is associated with poor prognosis (Alizadeh et al., 2000). Moreover, it was recently found that DLBCL can be grouped according to genomic alterations with different prognoses, thus reflecting the obvious heterogeneity of DLBCL (Schmitz et al., 2018). The standard chemotherapy treatment for DLBCL is rituximab plus cyclophosphamide, vincristine, doxorubicin and prednisone (R-CHOP) (Coiffier et al., 2002), but for 40%–45% of patients, this treatment is ineffective or relapse occurs soon after remission (Vitolo and Novo, 2021). Moreover, only 10%–20% of patients who relapse after first-line treatment can achieve long-term disease-free survival with salvage therapy, such as high-dose chemotherapy, stem cell transplantation and chimeric antigen receptor (CAR) T-cell therapy, and the median overall survival is only 5–6 months (Goy et al., 2019). However, the definite mechanism underlying disease relapse and drug resistance under the current standard therapy is still largely unknown. Therefore, overcoming the recurrence and drug resistance of DLBCL is the most important and difficult problem in current research.

Centrimeric protein F (CENPF) is a centromeric protein containing 3210 amino acids that plays an important role in mitosis, including centromere maturation, chromosome adjustment and segregation, and stabilization of the anaphase spindle, thereby regulating mitosis and maintaining the normal progression of the cell cycle (Varis et al., 2006). In addition to its involvement in tumor cell proliferation, CENPF is also involved in protein degradation in tumor cells (Gurden et al., 2010). The expression of CENPF in GO/Gl-cells is low, and it accumulates in the nuclear matrix during S-phase, with the maximum level in G2/M-cells (Liao et al., 1995). At present, CENPF is generally considered a potential marker of proliferation in a variety of malignant tumors, as it is elevated in various malignant tumors, such as liver cancer (Huang et al., 2021), gastric carcinoma (Chen et al., 2019), lung cancer (Li et al., 2021), breast cancer (Chen et al., 2020), prostate cancer (Shahid et al., 2019) and malignant glioma (Zhang et al., 2021) and is associated with poor prognosis in patients. In 2005, a radio-immunological assay was used to compare 347 non-Hodgkin’s lymphoma patients and 150 control subjects, and the proportions of CENPF detected in the two groups were 7.2% and 1.3% (*p* < 0.05), respectively, suggesting that CENPF may play a role in the development and progression of some types of NHL (Bencimon et al., 2005). However, the relationship between CENPF and DLBCL is not well reported, and the specific mechanism is unknown.

Ubiquitination (UB) is a protein posttranslational modification process characterized by the covalent attachment of ubiquitin molecules to cellular protein substrates through an enzymatic cascade, which involves E1 (ubiquitin-activating enzyme), E2 (ubiquitin-conjugating enzyme) and E3 (ubiquitin ligase) (Lacoursiere et al., 2022). The process of ubiquitination includes classification of intracellular proteins and selection of target protein molecules to undergo specific modifications. The ubiquitination modification process is reversible and dynamic and participates in the regulation of gene transcription, the cell cycle, inflammatory responses, DNA damage repair and other biological processes that mediate the occurrence and development of tumors (Popovic et al., 2014). The role of protein ubiquitination in malignant lymphoma has been extensively confirmed; non-etheless, our understanding of how protein ubiquitination regulates malignant lymphoma is still greatly limited. However, in our study, using DLBCL patient tissues and cell lines, we found that CENPF affected the immune microenvironment of DLBCL by altering ubiquitination modifications through proteomic and ubiquitinomic analyses, and we hope to identify the mechanism by which CENPF affects the prognosis of DLBCL and provide promising therapeutic strategies for DLBCL.

## Materials and methods

### Study population

This retrospective study was approved by the institutional review board, and written consent was obtained from involving patients. This study selected and reviewed 12 patients diagnosed with DLBCL in January 2021 at Nanjing First Hospital and completed six cycles of R-CHOP regimen chemotherapy, six of whom achieved complete remission and the other six were patients with refractory relapse.

### Reagents and cell lines

TRIzol reagent was purchased from Thermo Fisher Scientific (MA, United States), and the reverse transcription system kit and SYBR Premix Ex Taq II kit were obtained from Takara Biomedical Technology (Beijing, China). The CENPF antibody was purchased from Proteintech (Chicago, IL, United States). The primer sequences for CENPF and β-actin were synthesized and provided by Invitrogen (CA, United States). The DLBCL cell line SU-DHL-4 was purchased from American Type Culture Collection (ATCC, Bethesda, MD, United States) and cultured in RPMI-1640 medium (HyClone, UT, United States) with 10% fetal bovine serum (FBS; Sigma, St, Louis, MO, United States) at 37°C and 5% CO_2_.

### Adenovirus transfection

An adenovirus carrying CENPF overexpression plasmids was constructed by OBIO technology and transfected into a DLBCL cell line according to the manufacturer’s instructions. Approximately 3×10^3^ DLBCL cells were cultured in 96-well culture plates, and 100 µl of medium was added to each well. When the cells reached 60% confluency, the medium was changed to serum-free medium, and the corresponding volumes of adenovirus were added to each well according to the MOI (multiplicity of infection) value for transfection. After transfection for 8–12 h, the transfection efficiency was evaluated under a fluorescence microscope. Then, the cell samples were gently aspirated and centrifuged at 1000 rpm for 5 min, complete medium was added, and the culture was continued for 24–48 h.

### Real-time RT PCR

Total RNA was extracted from the lymphoma tissue using TRIzol reagent, and cDNA was prepared with a reverse transcription system kit. Then, real-time RT PCR was performed to evaluate the relative mRNA levels of genes in different lymphoma tissues by the SYBR Premix Ex Taq II kit in this study. Additionally, the mRNA expression levels were determined by the comparative 2^−ΔΔCt^ method. β-actin was used as the internal control in this study.

### Immunohistochemical staining

Immunohistochemical staining was performed in this study according to the protocol described previously (Pichaiwong et al., 2013). Briefly, the lymphoma tissue was cut into 4 μm slices, and the sections were incubated with primary antibodies overnight. Then, the secondary antibodies were used and visualized with diaminobenzidine under a microscope. More than 6 fields in each section were evaluated, and the IOD/area value was used to measure the intensity of positive staining using Image-Pro Plus 7.0 software (Media Cybernetics, Silver Spring, MD).

### Proteome and ubiquitome analyses

#### Sample preparation

The DLBCL cell line SU-DHL-4 was transfected with a CENPF-overexpressing adenovirus, and the samples were lysed with lysis buffer (8 M urea, 1% protease inhibitor and 50 μM PR-619). After ultrasonic lysis, the sample protein was centrifuged at 12000×*g* for 10 min to remove cellular debris. Furthermore, the supernatant was transferred into a centrifuge tube, and the protein concentration was determined using a BCA assay kit (Beyotime Biotechnology, Jiangsu, China). Proteome and ubiquitome analyses were performed at PTM BioLabs (Hangzhou, China).

### LC-MS/MS analysis

Equal amounts of sample protein were used for enzymatic digestion, and the volume was adjusted to be the same as the lysis solution. Then, TCA was slowly added to reach a proportion of 20%, and the mixture was incubated for 2 h at 4°C after vortex mixing. After digestion with trypsin at 37°C overnight, the peptides were collected. Dithiothreitol (DTT) and iodoacetamide (IAA) were added successively. Then, the peptides were further separated by high-performance liquid chromatography (HPLC, Thermo Fisher Scientific). The separated peptides were dissolved in IP buffer (pH 8.0, 1 mM EDTA, 50 mM Tris-HCl, 100 mM NaCl and 0.5% NP-40). Peptide adsorption resin (PTM-1104, PTM Bio) and 0.1% trifluoroacetic acid eluent were used to obtain the ubiquitinated peptides. Then, the peptide fractions were analyzed by LC‒MS/MS. The MS data were acquired dynamically based on the data-dependent top 10 method.

### Bioinformatics data analysis

Raw MS/MS data were further analyzed using Andromeda engine in the MaxQuant environment. The human UniProt FASTA database was used in this study and matched with the MS/MS spectra with FDR<1%. Categorical annotation was used and presented as molecular function and cellular component, Gene Ontology (GO) biological process and Kyoto Encyclopedia of Genes and Genomes (KEGG) pathways. GO and KEGG pathway enrichment analyses were performed based on Fisher’s test. Timer 2.0 (http://timer.cistrome.org/) was used to estimate immune infiltration in the environment of The Cancer Genome Atlas (TCGA) in this study (Li et al., 2020).

### Statistical analysis

The experiments were performed in biological triplicates, and the data were analyzed using SPSS 20.0 (Chicago, IL, United States) and are presented as the mean ± standard deviation. The correlation was determined by Pearson’s r, and the analysis between two groups was performed using Student’s unpaired t test or one-way analysis of variance. *p* < 0.05 was considered statistically significant in this study.

## Results

### CENPF overexpression was found in DLBCL patients with relapsed or refractory disease

**FIGURE 1:**
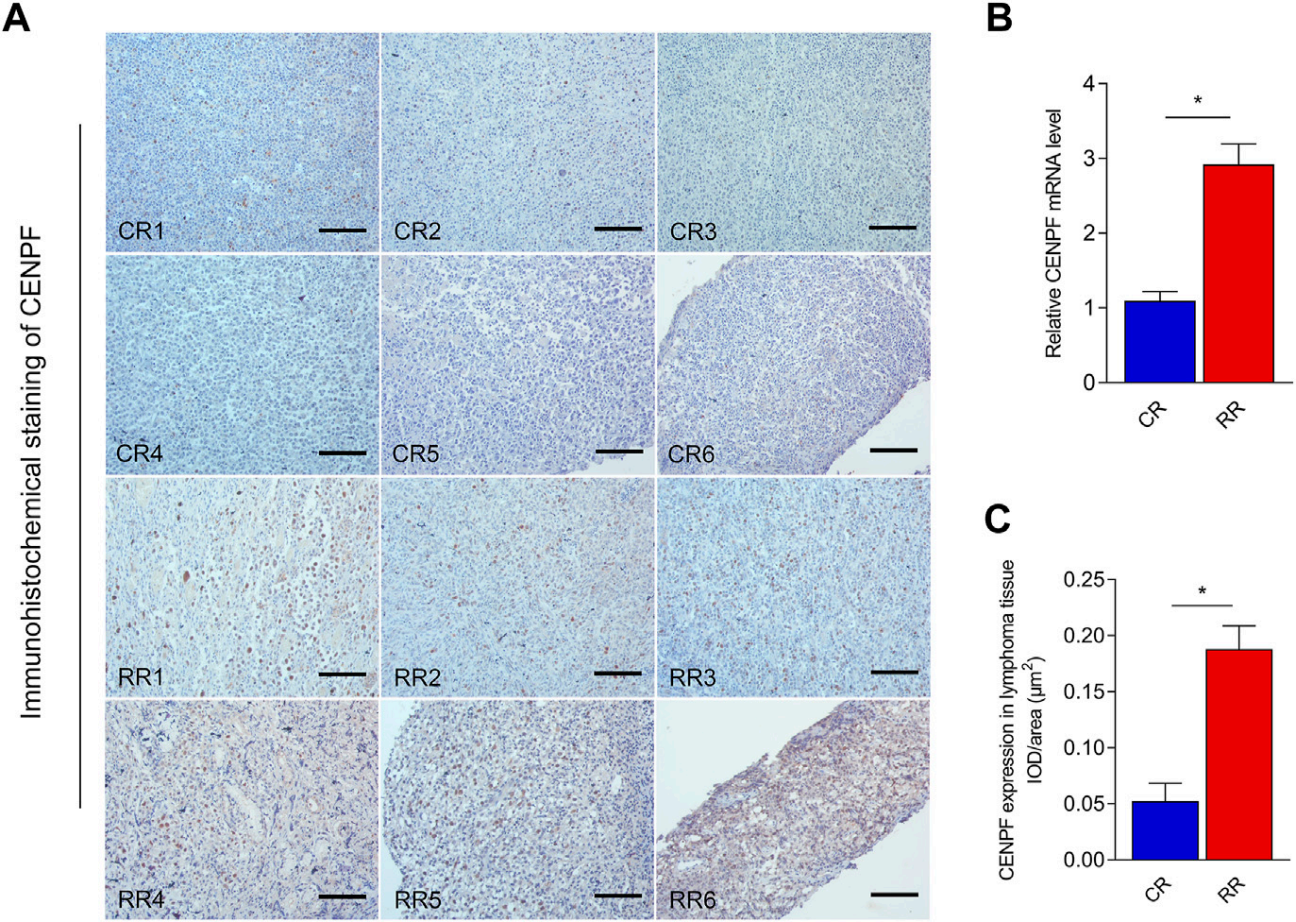
Real-time RT‒PCR and immunohistochemical staining analysis of CENPF expression in lymphoma **(A)**. Immunohistochemical staining of CENPF in CR and RR patients with lymphoma. **(B)**. Real-time RT PCR was performed to reveal the mRNA expression levels of CENPF in complete remission (CR) and relapsed refractory (RR) patients with lymphoma. **(C)**. Quantitative analysis of CENPF expressed in lymphoma tissue in different CR and RR patients by using the mean IOD/area (μm^2^). Scale bar is 200 μm.

Real-time PCR was first performed to reveal the expression levels of CENPF in patients with complete remission (CR) and relapsed refractory (RR) disease. As shown in Figure 1, in DLBCL patients, CENPF expression was significantly upregulated in RR patients compared with CR patients. Then, immunohistochemical staining of CENPF in lymphoma tissue was also performed to evaluate the expression and distribution of CENPF in CR patients and RR patients. The results showed that CENPF was overexpressed in RR patients, and the IOD/area measurement results were also consistent with the real-time PCR results in this study.

### Quantitative proteomic profiling of the CENPF-upregulated DLBCL cell line

**FIGURE 2:**
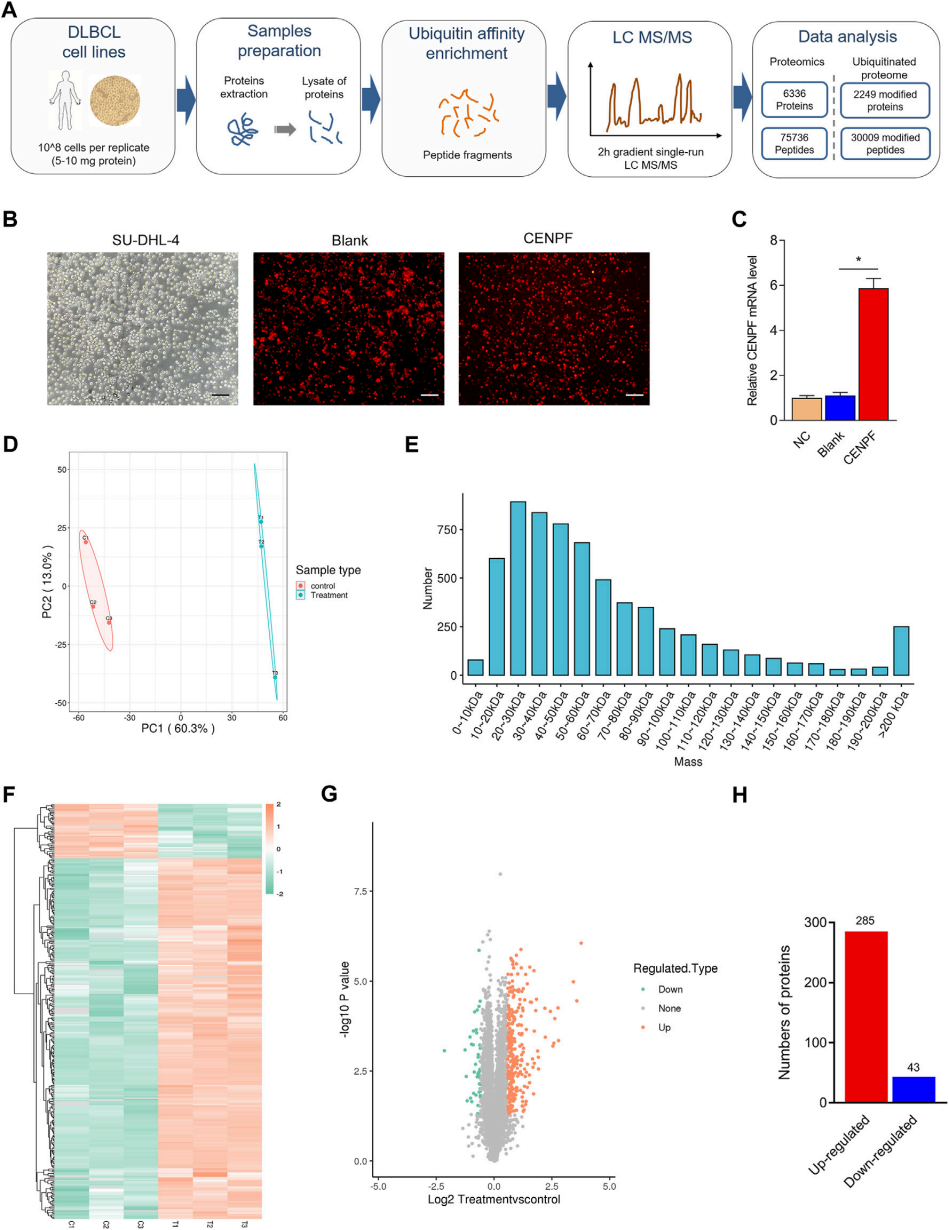
Quantitative proteomic profiling analysis in CENPF-upregulated DLBCL cells **(A)**. Flowcharts of the quantitative proteome and ubiquitinome analysis in CENPF-overexpressing DLBCL cells; **(B)**. The expression of CENPF in a DLBCL cell line (SU-DHL-4) was upregulated using an adenovirus and evaluated under a fluorescence microscope; scale bar is 100 μm; **(C)**. Quantitative analysis of CENPF expression in different groups of SU-DHL-4 cells; **(D)**. The results of principal component analysis in the control group and CENPF-upregulated group; **(E)**. The numbers of proteins with different molecular masses determined by proteome analysis in DLBCL cells; f-g. The results of clustering analysis by proteome analysis are presented as a heatmap and volcano plots (log2-fold change and–log10 *p* value). **(H)**. The numbers of upregulated and downregulated proteins identified by quantitative proteomics.

To systematically identify the role of overexpressed CENPF in DLBCL, a DLBCL cell line (SU-DHL-4) was used, and mass spectrometry-based quantitative proteomics was performed in this study (Figure 2A). CENPF expression was first determined in the SU-DHL-4 cell line, and the results demonstrated that CENPF expression was not activated in the normal DLBCL cell line. Then, adenovirus was used to upregulate CENPF in the human SU-DHL-4 cell line, and quantitative proteomic analysis was performed according to a protocol described previously. As shown in Figures 2B,C, transfection was performed using an adenovirus, and CENPF was found to be significantly increased in the human DLBCL cell line. Then, quantitative proteomics was performed, and the principal component analysis (PCA) results were obviously different after CENPF was upregulated (Figure 2D). The expression levels of various proteins were also markedly changed in the DLBCL cell line and were mainly concentrated in the small- and medium-sized protein population of 10–100 kDa (Figure 2E). A total of 6336 proteins and 75736 peptides were identified in the DLBCL cell line by quantitative proteomics, and 328 proteins were considered evidently changed after CENPF upregulation based on a fold ratio >1.5. The significantly changed proteins included 285 upregulated proteins and 43 downregulated proteins. The results of the clustering analysis are presented as heatmaps (Figure 2F) and volcano plots (Figure 2G, log2-fold change and–log10 *p* value), which demonstrated that the expression levels of most proteins were evidently upregulated. The upregulated proteins accounted for 86.9% of all the significantly changed proteins.

### Functional annotation of proteins in CENPF-overexpressing DLBCL cells

**FIGURE 3:**
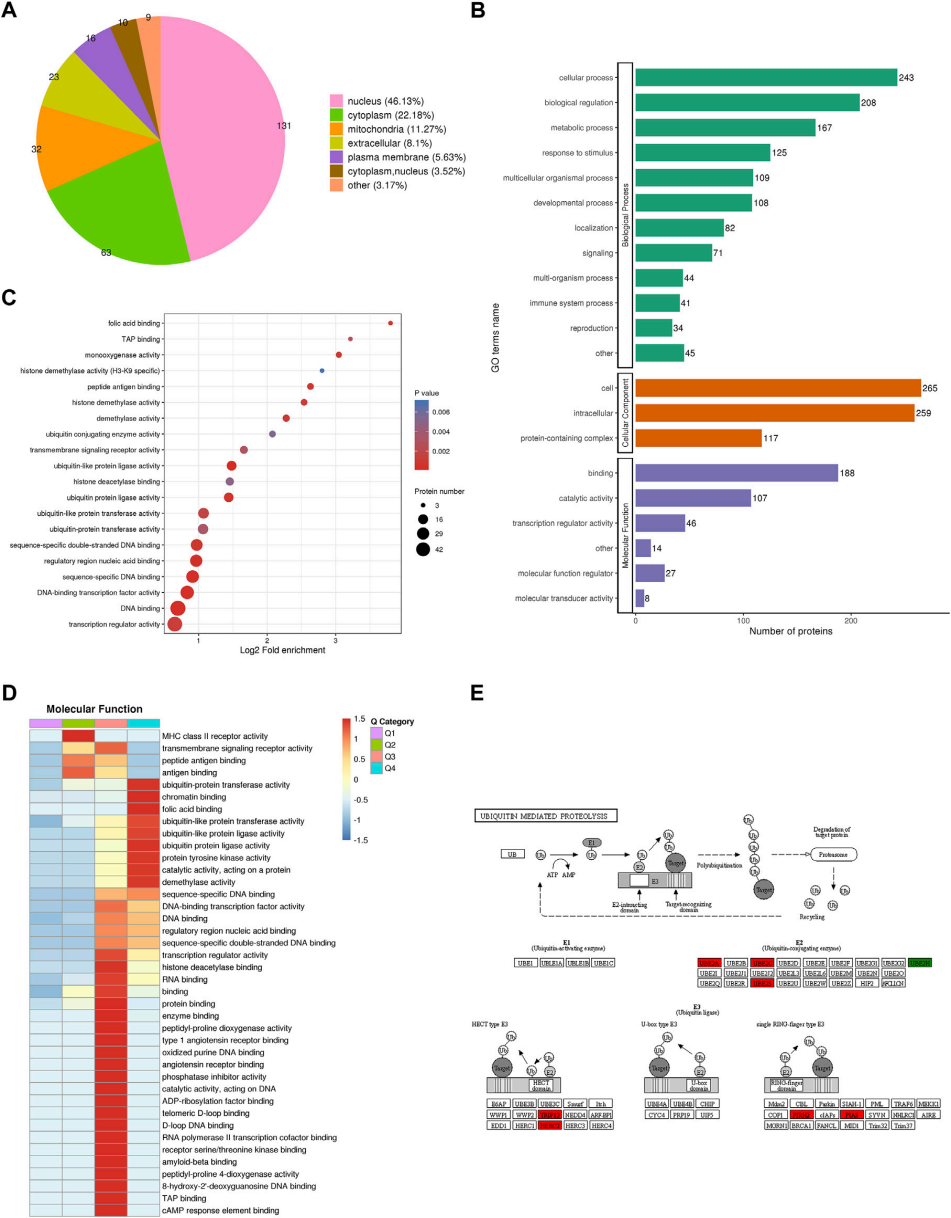
Bioinformatics analysis and protein functional annotation. **(A)**. The subcellular localization of proteins identified by quantitative proteomics in CENPF-upregulated DLBCL cells; **(B)**. Gene Ontology (GO)-based enrichment analysis of proteins with significantly changed expression levels; **(C,D)**. The GO analysis and COG/KOG functional classification of proteins in the proteome; **(E)**. The significantly increased proteins in ubiquitin-mediated proteolysis, including UBE2A, UBE2C, UBE2S, TRIP12, HERC2, PIRH2 and PIAS.

To reveal the function of proteins identified by quantitative proteomics in CENPF-upregulated DLBCL cells, subcellular localization, gene ontology (GO)-based enrichment analysis, Kyoto Encyclopedia of Genes and Genomes (KEGG) pathway enrichment, protein domain analysis and COG/KOG functional classification were performed. As shown in Figure 3A, WolF Psort software was first used to annotate the subcellular structures of significantly changed proteins, and then localization to various intracellular elements was determined based on the differences in the membrane structure of the proteins in eukaryotic tissue cells. The results demonstrated that the significantly changed proteins were mostly localized in the nucleus (46.13%), cytoplasm (22.18%) and mitochondria (11.27%). Additionally, the results of GO analysis were presented as biological process, cellular component and molecular function terms, and the proteins identified by GO analysis were mainly focused in cellular processes, biological regulation, immune system processes and transcription regulator activity. Furthermore, the significantly increased proteins (fold ratios >2.0) identified in this study were mainly enriched in various ubiquitin-related kinase activities, which was revealed by functional enrichment analysis, as shown in Figures 3C, D. The proteins and kinases found to be significantly increased, including UBE2A, UBE2C, UBE2S, TRIP12, HERC2, PIRH2 and PIAS, were closely associated with the ubiquitination process. The proteins participating in the process of ubiquitin-mediated proteolysis are presented in Figure 3E.

### Ubiquitinome analysis in CENPF-upregulated DLBCL cells

**FIGURE 4:**
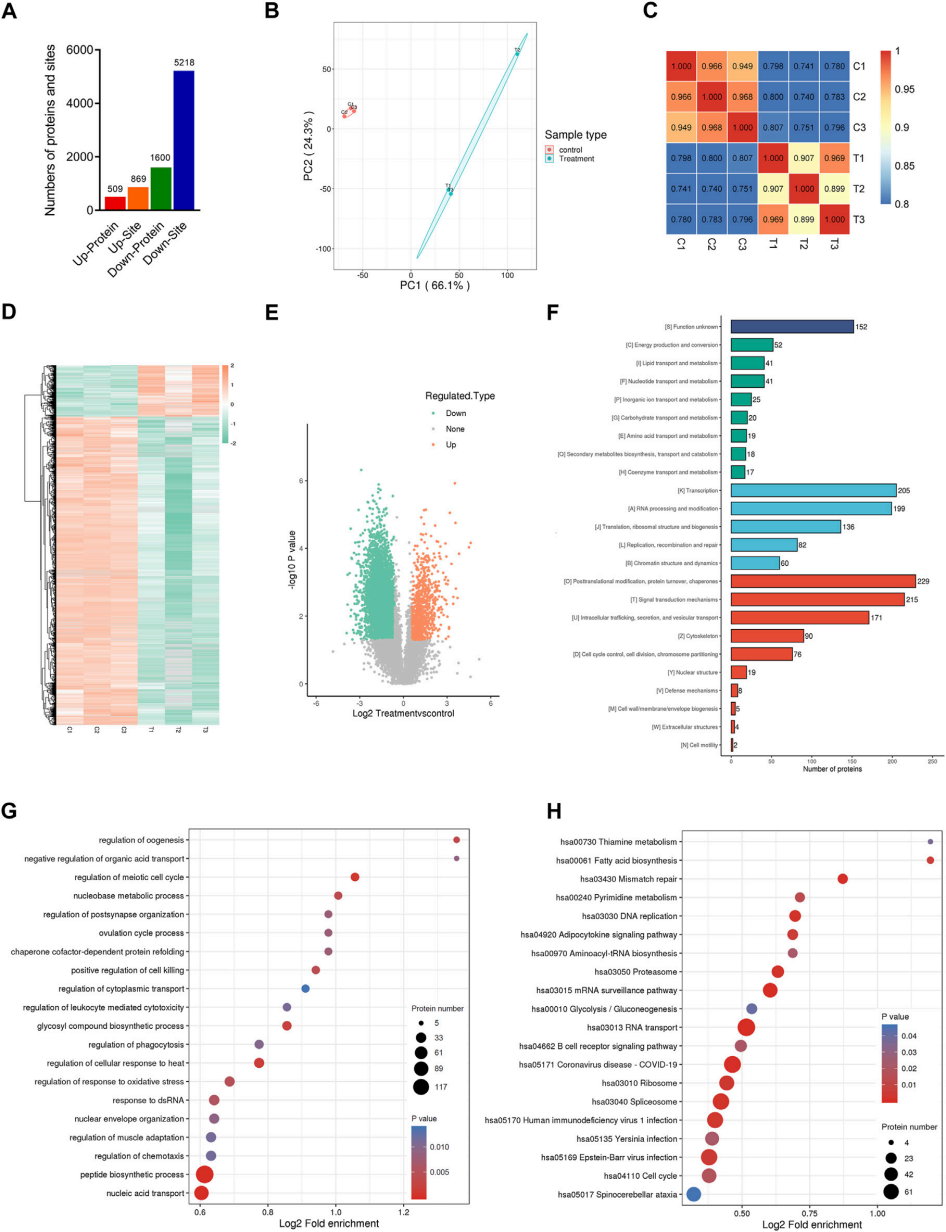
Quantitative ubiquitinome analysis in CENPF-upregulated DLBCL cells **(A)**. The numbers of proteins and sites identified by ubiquitinome analysis in this study; **(B)**. The results of principal component analysis in the control group and CENPF-upregulated group identified by ubiquitinome; **(C)**. Pearson’s correlation coefficient analysis performed in different groups; **(D,E)**. The results of clustering analysis by ubiquitinome analysis are presented as a heatmap and volcano plots (log2-fold change and–log10 *p* value); **(F)**. GO analysis and COG/KOG functional classification of proteins identified by ubiquitinome analysis; **(G,H)**. The results of functional enrichment and KEGG enrichment analysis.

Previous results showed that the activities of multiple kinases associated with the ubiquitination process were significantly altered in CENPF-overexpressing DLBCL cells. Thus, quantitative analysis of the ubiquitinome was further performed in this study. As shown in Figure 4A, CENPF-upregulated DLBCL cells were quantitatively analyzed by the ubiquitinome, and a total of 2249 ubiquitination proteins and 30582 peptides were identified in DLBCL cells in this study. The proteins and sites included 587 upregulated ubiquitination proteins, 1000 upregulated ubiquitination sites, 1662 downregulated ubiquitination proteins and 5451 downregulated ubiquitination sites. The results showed that the proteins in DLBCL cells were markedly deubiquitinated after CENPF activation in this study. Principal component analysis significantly distinguished between the CENPF upregulated group and the control group (Figure 4B). Pearson’s correlation coefficient analysis also demonstrated a significant correlation between different samples within a certain group. Additionally, as shown in Figures 4D, E, the clustering analysis presented as heatmaps (Figure 4D) and volcano plots (Figure 4E, log2-fold change and–log10 *p* value) demonstrated that the degree of protein ubiquitination was significantly decreased in DLBCL cells after CENPF overexpression. Most proteins were in abnormal ubiquitination or deubiquitination states (73.9%). Additionally, GO and KEGG pathway analyses were performed to uncover the underlying molecular mechanisms in which the proteins participated. The results of COG/KOG functional analysis demonstrated that these proteins were mainly associated with transcription, signal transduction and posttranslational modification. The proteins were significantly enriched in the regulation of the meiotic cell cycle, chaperone cofactor-dependent protein refolding and regulation of phagocytosis. The results of KEGG pathway analysis also demonstrated enrichment of immune-associated pathways, such as the B-cell receptor signaling pathway.

### CENPF overexpression mediated protein deubiquitination in various immune pathways

**FIGURE 5:**
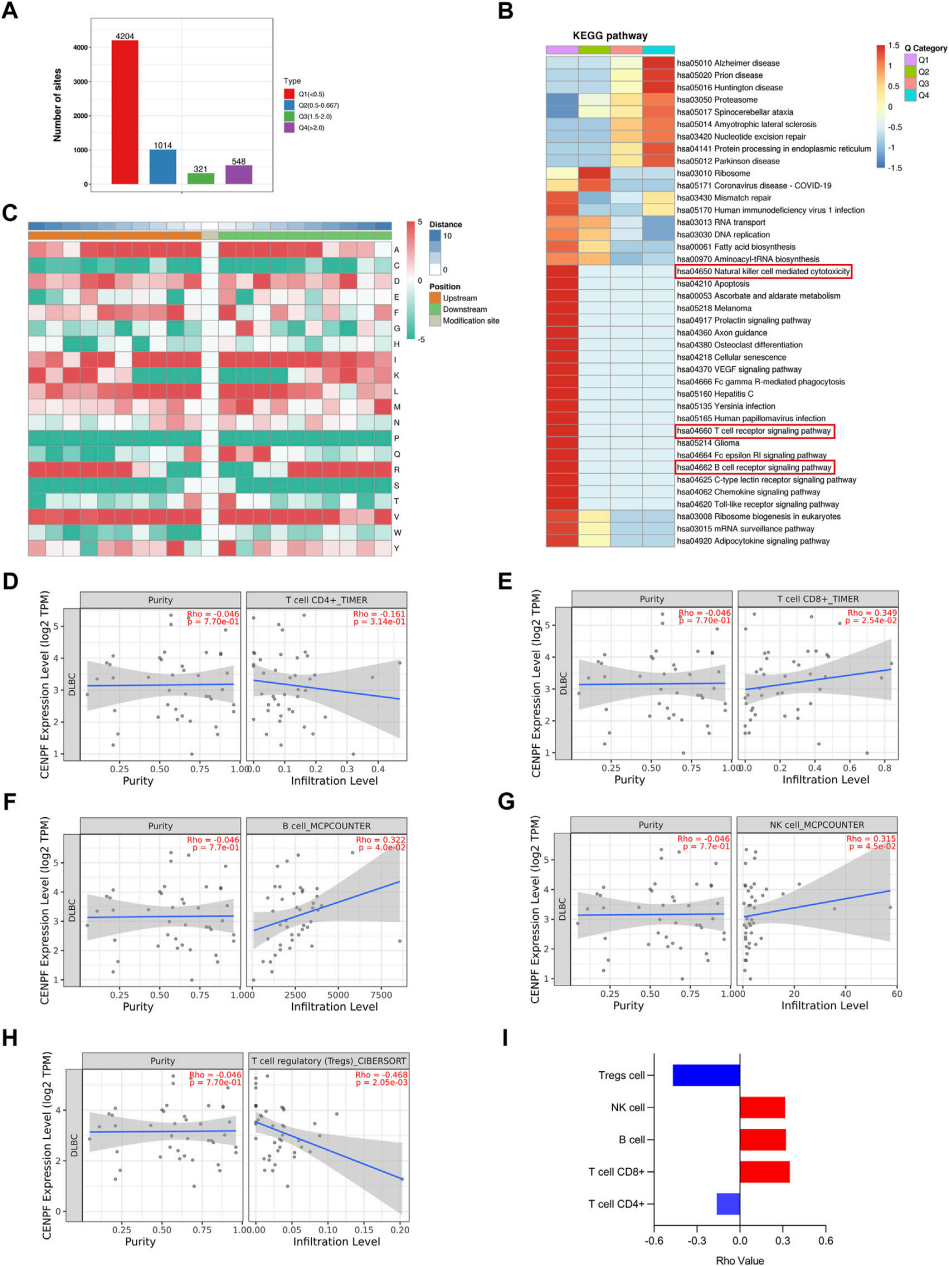
Bioinformatics analysis and immune infiltration evaluation in DLBCL cells **(A)**. The ubiquitination sites were divided into 4 parts according to the fold change in ubiquitination levels in CENPF-upregulated DLBCL cells: Part Q1 (fold change <0.5), Part Q2 (fold change from 0.5 to 0.667), Part Q3 (fold change from 1.5 to 2.0) and Part Q4 (fold change >2.0); **(B)**. The results of KEGG pathway enrichment analysis of proteins identified by ubiquitinome; **(C)**. The motif analysis of ubiquitination sites in this study; d-i. The role of CENPF in immune infiltration in DLBCL presented with CD4^+^ T cells, CD8^+^ T cells, B cells, NK cells and regulatory T cells verified using TIMER2.0.

To further analyze the proteins with significantly changed ubiquitination in this study, the proteins and sites were further divided into 4 parts from Q1 to Q4 according to the fold change in ubiquitination levels in CENPF-upregulated DLBCL cells. As shown in Figure 5A, there were 4204 sites in Part Q1 (folds <0.5, 69.1%), 1014 sites in Part Q2 (from 0.5 to 0.667, 16.6%), 321 sites in Part Q3 (from 1.5 to 2.0, 5.27%) and 548 sites in Part Q4 (>2.0, 9.0%). The results demonstrated that the level of ubiquitination at most sites was significantly decreased in DLBCL cells after CENPF overexpression. Then, the GO classification, KEGG pathway and protein domain enrichment analyses were performed according to the different parts from Q1 to Q4, and cluster analysis was performed to find the correlation of protein functions in different signaling pathways. As shown in Figure 5B, the significantly deubiquitinated proteins and sites were evidently enriched in many immune-related cellular processes and pathways, including natural killer cell-mediated cytotoxicity, the T-cell receptor signaling pathway and the B-cell receptor signaling pathway. Additionally, pathways such as apoptosis, cellular senescence and Toll-like receptor signaling pathways were also enriched in Part Q1.

Furthermore, the TIMER2.0 database was used to verify the role of CENPF in immune infiltration in DLBCL and showed that CENPF is related to CD4^+^ T cells, CD8^+^ T cells, B cells, NK cells and regulatory T cells. In DLBCL patients, CENPF expression was positively correlated with CD8^+^ T cells, NK cells and B lymphocytes but negatively correlated with regulatory T cells. Although not statistically significant, a negative correlation between CENPF expression and CD4^+^ T cells was observed.

## Discussion

DLBCL, one of the most common and aggressive types of B-cell lymphoma, is heterogeneous; although more than half of DLBCL patients can be cured with standard treatment, it remains incurable in 40%–45% of patients (Vitolo and Novo, 2021). R/R DLBCL remains a major cause of morbidity and mortality, but the mechanism is still unclear. In our study, we retrospectively collected lymphoma tissues from DLBCL patients with refractory or relapsed (R/R) disease and complete remission (CR) and found that in the two groups, CENPF expression was higher in the R/R patients by real-time PCR and immunohistochemical staining, which demonstrated that CENPF overexpression may be associated with poor outcome in DLBCL patients. CENPF has recently been investigated as a centromeric protein that is highly expressed in a variety of tumors, including DLBCL, and has been shown to be an important promoter and master regulator of tumors associated with poor prognosis. In terms of the mechanism, it has been found that CENPF plays a role in breast cancer through activation of the PI3K-AKT-mTORC1 pathway (Sun et al., 2019). In prostate cancer (PC), CENPF has been reported to be a critical regulator of PC metabolism (Shahid et al., 2019). Also, knockdown of CENPF can inhibit the progression of lung adenocarcinoma by suppressing the expression of ER2/5 of the ERβ2/5 pathway (Hexiao et al., 2021). However, the mechanism of how CENPF acts in DLBCL is not yet clear; thus, we performed quantitative proteomic profiling of the CENPF-upregulated DLBCL cell line (generated by adenoviral transfection) and found that the expression levels of various proteins in the DLBCL cell line were also markedly changed, and these proteins were mainly focused on cellular processes, biological regulation, immune system processes and transcriptional regulator activity by GO and KEGG analysis. Furthermore, it was interesting that the significantly increased proteins were mainly enriched with various ubiquitination-related enzymes, which was revealed by functional enrichment. This finding was consistent with previous findings that CENPF participates in ubiquitin-mediated proteolysis in cancer (Shi and Zhang, 2017; Zhang et al., 2021).

Ubiquitination (UB) is a protein posttranslational modification process that is strongly correlated with the development and prognosis of DLBCL. In ABC DLBCL, protein polyubiquitination has been considered to be a significant oncogenic signaling pathway based on the observation that inactivation of deubiquitinating enzyme (DUB) A20 and mutation of TRAF2 both result in activation of NF-κB (Compagno et al., 2009; Honma et al., 2009). In addition, protein modification with linear ubiquitin chains (LUBAC) plays a crucial role in the oncogenic activation of NF-κB in ABC DLBCL cells because LUBAC attaches linear polyubiquitin chains to IκB kinase-γ, an indispensable element for the function of NF-κB (Yang et al., 2014). Proteins of the F-box family act as specific subunits in the ubiquitin ligase complex of Skp1–Cul1–F-box-protein specificity (SCF), selectively guiding SCF to recognize target proteins and promote their enzymatic digestion after ubiquitination. FBXO10 and FBXO11 of the FBXO family were recently identified as important regulators of DLBCL. They both play a role in inhibiting DLBCL tumors. FBXO10 and FBXO11 bind to BCL2 and BCL6, respectively, resulting in their ubiquitination and promoting their degradation, thereby regulating the levels of BCL2 and BCL6 proteins in lymphoma cells (Duan et al., 2012; Chiorazzi et al., 2013). Additionally, in our study, the results showed that the proteins in DLBCL cells were markedly deubiquitinated after CENPF activation. Therefore, the protein ubiquitination system has been shown to be closely related to the development of DLBCL, and it is expected to become a therapeutic target for malignant lymphoma, but we need further research on how to target this mechanism with small molecules.

Studies have found that the degree of infiltration of immune cells influences OS in DLBCL patients, indicating that immune cells are closely associated with the prognosis and treatment of DLBCL (Liang et al., 2021). The ubiquitinated modification pathway undoubtedly also plays an important role in the regulation of the immune system, and it has an effect on intrinsic immunity and adaptive immunity by regulating the function of different types of cells in the immune system, thereby affecting the occurrence and development of many major human diseases, such as autoimmune diseases, infectious diseases and malignant tumors (Gavali et al., 2021; Çetin et al., 2021). Meanwhile, we found that after the overexpression of CENPF, the proteins identified by quantitative proteomics in CENPF-upregulated DLBCL cells were mainly associated with transcription, signal transduction and posttranslational modification through COG/KOG functional analysis. The results of KEGG pathway analysis also demonstrated enrichment of immune-associated pathways, such as the B-cell receptor signaling pathway. CENPs, such as CENPA and CENPB, have been discovered to be immune autoantigens of systemic scleroderma (Nunes et al., 2018). In addition, studies have found that CENPF is positively associated with CD4^+^ memory T-cell phenotypic markers and negatively correlated with memory T-cell survival regulators in cutaneous melanoma (Li et al., 2022). Therefore, CENPF may be related to the activation of memory CD4^+^ T cells, and the increased expression level of CENPF contributes to immunosuppression by restraining the maintenance and homeostasis of memory CD4^+^ T cells (Li et al., 2022). In the peripheral blood of hepatocellular carcinoma patients, the expression of CENPF was upregulated and was found to be positively correlated with the percentage of CD8^+^ T cells and negatively correlated with the percentage of CD4^+^ T cells (Si et al., 2021). In our study, we found that high CENPF expression alters the levels of ubiquitinating enzymes, which were mainly enriched in immune regulation. This result was consistent with a previous finding that CENPF may be associated with immune regulation. We further found that CENPF expression was positively correlated with CD8^+^ T cells in DLBCL samples. Although not statistically significant, a negative trend of correlation between CENPF expression and CD4^+^ T cells was observed; we propose that the lack of statistical significance may be related to the small sample size and the lack of further phenotypic analysis. We also found that the expression of CENPF was positively correlated with NK cells and B lymphocytes and negatively correlated with regulatory T cells. Therefore, further studies are needed to determine whether CENPF expression affects immune cell infiltration and tumor escape.

In conclusion, we believe that the role of CENPF in relapsed refractory DLBCL is related to the prognosis of patients. Notably, high expression of CENPF causes alterations in the activity of multiple ubiquitinating enzymes, and these enzymes are mainly enriched in immune-related pathways. We found that CENPF was positively correlated with CD8^+^ T cells, NK cells and B lymphocytes in DLBCL, while it tended to be negatively correlated with CD4^+^ T cells, so we suggest that CENPF may induce immune dysregulation by mediating protein deubiquitination in multiple immune pathways. Meanwhile, the current study confirmed that immunomodulation plays an important role in the development of DLBCL, especially immunotherapy, such as CAT-T-cell therapy, which has improved the prognosis of patients. Therefore, CENPF may be a potential clinical prognostic marker for DLBCL and an effective therapeutic target, but further studies are needed to validate these findings and clarify the mechanisms involved.

## Data Availability

The supplementary data presented in the study are included in the part of Supplementary Material, further inquiries can be provided by connecting with the corresponding authors.

## References

[B1] Alizadeh A. A. Eisen M. B. Davis R. E. Ma C. Lossos I. S. Rosenwald A. (2000). Distinct types of diffuse large B-cell lymphoma identified by gene expression profiling. Nature 403, 503–511. 10.1038/35000501 10676951

[B2] Bencimon C. Salles G. Moreira A. Guyomard S. Coiffier B. Bienvenu J. (2005). Prevalence of anticentromere F protein autoantibodies in 347 patients with non-Hodgkin's lymphoma. Ann. N. Y. Acad. Sci. 1050, 319–326. 10.1196/annals.1313.034 16014548

[B3] Cazzola M. (2016). Introduction to a review series: The 2016 revision of the WHO classification of tumors of hematopoietic and lymphoid tissues. Blood 127, 2361–2364. 10.1182/blood-2016-03-657379 27069255

[B4] Çetin G. Klafack S. Studencka-Turski M. Krüger E. Ebstein F. (2021). The ubiquitin-proteasome system in immune cells. Biomolecules 11, 60. 10.3390/biom11010060 33466553 PMC7824874

[B5] Chen E. B. Qin X. Peng K. Li Q. Tang C. Wei Y. C. (2019). HnRNPR-CCNB1/CENPF axis contributes to gastric cancer proliferation and metastasis. Aging (Albany NY) 11, 7473–7491. 10.18632/aging.102254 31527303 PMC6782008

[B6] Chen Q. Xu H. Zhu J. Feng K. Hu C. (2020). LncRNA MCM3AP-AS1 promotes breast cancer progression via modulating miR-28-5p/CENPF axis. Biomed. Pharmacother. 128, 110289. 10.1016/j.biopha.2020.110289 32485570

[B7] Chiorazzi M. Rui L. Yang Y. Ceribelli M. Tishbi N. Maurer C. W. (2013). Related F-box proteins control cell death in *Caenorhabditis elegans* and human lymphoma. Proc. Natl. Acad. Sci. U. S. A. 110, 3943–3948. 10.1073/pnas.1217271110 23431138 PMC3593917

[B8] Coiffier B. Lepage E. Briere J. Herbrecht R. Tilly H. Bouabdallah R. (2002). CHOP chemotherapy plus rituximab compared with CHOP alone in elderly patients with diffuse large-B-cell lymphoma. N. Engl. J. Med. 346, 235–242. 10.1056/NEJMoa011795 11807147

[B9] Compagno M. Lim W. K. Grunn A. Nandula S. V. Brahmachary M. Shen Q. (2009). Mutations of multiple genes cause deregulation of NF-kappaB in diffuse large B-cell lymphoma. Nature 459, 717–721. 10.1038/nature07968 19412164 PMC2973325

[B10] Duan S. Cermak L. Pagan J. K. Rossi M. Martinengo C. di Celle P. F. (2012). FBXO11 targets BCL6 for degradation and is inactivated in diffuse large B-cell lymphomas. Nature 481, 90–93. 10.1038/nature10688 22113614 PMC3344385

[B11] Gavali S. Liu J. Li X. Paolino M. (2021). Ubiquitination in T-cell activation and checkpoint inhibition: New avenues for targeted cancer immunotherapy. Int. J. Mol. Sci. 22, 10800. 10.3390/ijms221910800 34639141 PMC8509743

[B12] Goy A. Ramchandren R. Ghosh N. Munoz J. Morgan D. S. Dang N. H. (2019). Ibrutinib plus lenalidomide and rituximab has promising activity in relapsed/refractory non-germinal center B-cell-like DLBCL. Blood 134, 1024–1036. 10.1182/blood.2018891598 31331917 PMC6764267

[B13] Gurden M. D. Holland A. J. van Zon W. Tighe A. Vergnolle M. A. Andres D. A. (2010). Cdc20 is required for the post-anaphase, KEN-dependent degradation of centromere protein F. J. Cell Sci. 123, 321–330. 10.1242/jcs.062075 20053638 PMC2816182

[B14] Hexiao T. Yuquan B. Lecai X. Yanhong W. Li S. Weidong H. (2021). Knockdown of CENPF inhibits the progression of lung adenocarcinoma mediated by ERβ2/5 pathway. Aging (Albany NY) 13, 2604–2625. 10.18632/aging.202303 33428600 PMC7880349

[B15] Honma K. Tsuzuki S. Nakagawa M. Tagawa H. Nakamura S. Morishima Y. (2009). TNFAIP3/A20 functions as a novel tumor suppressor gene in several subtypes of non-Hodgkin lymphomas. Blood 114, 2467–2475. 10.1182/blood-2008-12-194852 19608751

[B16] Huang Y. Chen X. Wang L. Wang T. Tang X. Su X. (2021). Centromere protein F (CENPF) serves as a potential prognostic biomarker and target for human hepatocellular carcinoma. J. Cancer 12, 2933–2951. 10.7150/jca.52187 33854594 PMC8040902

[B17] Lacoursiere R. E. Hadi D. Shaw G. S. (2022). Acetylation, phosphorylation, ubiquitination (oh my!): Following post-translational modifications on the ubiquitin road. Biomolecules 12, 467. 10.3390/biom12030467 35327659 PMC8946176

[B18] Li M. X. Zhang M. Y. Dong H. H. Li A. J. Teng H. F. Liu A. L. (2021). Overexpression of CENPF is associated with progression and poor prognosis of lung adenocarcinoma. Int. J. Med. Sci. 18, 494–504. 10.7150/ijms.49041 33390818 PMC7757141

[B19] Li M. Zhao J. Yang R. Cai R. Liu X. Xie J. (2022). CENPF as an independent prognostic and metastasis biomarker corresponding to CD4+ memory T cells in cutaneous melanoma. Cancer Sci. 113, 1220–1234. 10.1111/cas.15303 35189004 PMC8990861

[B20] Li T. Fu J. Zeng Z. Cohen D. Li J. Chen Q. (2020). TIMER2.0 for analysis of tumor-infiltrating immune cells. Nucleic Acids Res. 48, W509–w514. 10.1093/nar/gkaa407 32442275 PMC7319575

[B21] Liang X. J. Fu R. Y. Wang H. N. Yang J. Yao N. Liu X. D. (2021). An immune-related prognostic classifier is associated with diffuse large B cell lymphoma microenvironment. J. Immunol. Res. 2021, 5564568. 10.1155/2021/5564568 34212052 PMC8205595

[B22] Liao H. Winkfein R. J. Mack G. Rattner J. B. Yen T. J. (1995). CENP-F is a protein of the nuclear matrix that assembles onto kinetochores at late G2 and is rapidly degraded after mitosis. J. Cell Biol. 130, 507–518. 10.1083/jcb.130.3.507 7542657 PMC2120529

[B23] Nunes J. P. L. Cunha A. C. Meirinhos T. Nunes A. Araújo P. M. Godinho A. R. (2018). Prevalence of auto-antibodies associated to pulmonary arterial hypertension in scleroderma - a review. Autoimmun. Rev. 17, 1186–1201. 10.1016/j.autrev.2018.06.009 30316987

[B24] Pichaiwong W. Hudkins K. L. Wietecha T. Nguyen T. Q. Tachaudomdach C. Li W. (2013). Reversibility of structural and functional damage in a model of advanced diabetic nephropathy. J. Am. Soc. Nephrol. 24, 1088–1102. 10.1681/asn.2012050445 23641056 PMC3699819

[B25] Popovic D. Vucic D. Dikic I. (2014). Ubiquitination in disease pathogenesis and treatment. Nat. Med. 20, 1242–1253. 10.1038/nm.3739 25375928

[B26] Schmitz R. Wright G. W. Huang D. W. Johnson C. A. Phelan J. D. Wang J. Q. (2018). Genetics and pathogenesis of diffuse large B-cell lymphoma. N. Engl. J. Med. 378, 1396–1407. 10.1056/NEJMoa1801445 29641966 PMC6010183

[B27] Shahid M. Kim M. Lee M. Y. Yeon A. You S. Kim H. L. (2019). Downregulation of CENPF remodels prostate cancer cells and alters cellular metabolism. Proteomics 19, e1900038. 10.1002/pmic.201900038 30957416 PMC6633900

[B28] Shi C. Zhang Z. (2017). Screening of potentially crucial genes and regulatory factors involved in epithelial ovarian cancer using microarray analysis. Oncol. Lett. 14, 725–732. 10.3892/ol.2017.6183 28693226 PMC5494615

[B29] Si T. Huang Z. Jiang Y. Walker-Jacobs A. Gill S. Hegarty R. (2021). Expression levels of three key genes CCNB1, CDC20, and CENPF in HCC are associated with antitumor immunity. Front. Oncol. 11, 738841. 10.3389/fonc.2021.738841 34660300 PMC8515852

[B30] Smith A. Crouch S. Lax S. Li J. Painter D. Howell D. (2015). Lymphoma incidence, survival and prevalence 2004-2014: Sub-type analyses from the UK's haematological malignancy research network. Br. J. Cancer 112, 1575–1584. 10.1038/bjc.2015.94 25867256 PMC4453686

[B31] Sun J. Huang J. Lan J. Zhou K. Gao Y. Song Z. (2019). Overexpression of CENPF correlates with poor prognosis and tumor bone metastasis in breast cancer. Cancer Cell Int. 19, 264. 10.1186/s12935-019-0986-8 31632198 PMC6788011

[B32] Varis A. Salmela A. L. Kallio M. J. (2006). Cenp-F (mitosin) is more than a mitotic marker. Chromosoma 115, 288–295. 10.1007/s00412-005-0046-0 16565862

[B33] Vitolo U. Novo M. (2021). Bcl-2 inhibition in DLBCL: “the times they are a-changing”. Blood 137, 577–579. 10.1182/blood.2020008924 33538802

[B34] Yang Y. Schmitz R. Mitala J. Whiting A. Xiao W. Ceribelli M. (2014). Essential role of the linear ubiquitin chain assembly complex in lymphoma revealed by rare germline polymorphisms. Cancer Discov. 4, 480–493. 10.1158/2159-8290.cd-13-0915 24491438 PMC3992927

[B35] Zhang M. Zhang Q. Bai J. Zhao Z. Zhang J. (2021). Transcriptome analysis revealed CENPF associated with glioma prognosis. Math. Biosci. Eng. 18, 2077–2096. 10.3934/mbe.2021107 33892537

